# Rheology for Wood Plastic Composite Extrusion—Part 1: Laboratory vs. On-Line Rheometry

**DOI:** 10.3390/polym17202782

**Published:** 2025-10-17

**Authors:** Krzysztof J. Wilczyński, Kamila Buziak, Adrian Lewandowski, Krzysztof Wilczyński

**Affiliations:** 1 Hanplast Ltd., 85-862 Bydgoszcz, Poland; wilczynski_k@wp.pl; 2 The Faculty of Mechanical and Industrial Engineering, Polymer Processing Institute, Warsaw University of Technology, 02-524 Warsaw, Poland; kamila.buziak@pw.edu.pl (K.B.); adrian.lewandowski@pw.edu.pl (A.L.)

**Keywords:** wood plastic composites, rheology, extrusion, viscosity

## Abstract

Common polymeric materials (neat polymers) are quite well known, and their properties are often available in appropriate material databases. However, material data, e.g., rheological data, for materials such as polymer blends, polymer composites (including wood plastic composites), and filled plastics are simply lacking in material databases. This paper addresses the problem of determining viscosity curves for one of the most widely used advanced polymeric materials: wood plastic composites. Studies were conducted in laboratory and production settings, i.e., on-line. Laboratory tests were conducted in two ways: on the basis of classical rheometric measurements, i.e., High-Pressure Capillary Rheometry (HPCR), and on the basis of Melt Flow Index (MFI) measurements, also including tests based on a limited number of measurement points. Tests in production conditions, i.e., on-line, were conducted during the extrusion process using the measurement of the process output (material flow rate) and pressure in a specialized extrusion die. The test results (viscosity curves) obtained from Melt Flow Index (MFI) measurements and on-line measurements were presented and evaluated against the background of the results (viscosity curves) obtained from classical capillary rheometry measurements (HPCR). Due to the lack of rheological data of wood plastic composites in available databases, in-house research methods based on the two-point viscosity curve determination in the plastometric (MFI) tests and the tests under production conditions, that is, on-line, have been proposed. The two-point method, based on the power law model, is quick and easy to implement, and allows for solving many polymer processing issues analytically. On-line tests have the significant advantage of being conducted under the actual flow conditions of the tested material, rather than under laboratory conditions, as is the case with rheometric and plastometric tests, which do not take into account the processing history of the tested material. The issues of rheology and modeling of wood plastic composite processing, e.g., extrusion and injection molding, which have not yet been resolved and require practical solutions, were also discussed. The results of this part of the study (viscosity curves and models) will be used in the second part of the study to evaluate the impact of rheological testing methods and rheological models on the accuracy of process modeling (extrusion).

## 1. Introduction

Knowledge of the material properties of polymeric materials, especially rheological properties, is the basis for the correct design of processing and effective and economic processing [1,2,3,4].

Rheological studies are difficult, time-consuming, and expensive. They are often conducted under conditions (temperature, pressure, and shear rate) that are distant from the actual processing conditions. For these reasons, simplified methods for determining rheological properties and methods implemented under processing conditions, i.e., on-line, are sought [2,3,5,6].

Common polymeric materials (neat polymers) are quite well known, and their properties are often available in appropriate material databases, e.g., CAMPUS [7], Autodesk Moldflow [8], Moldex-3D [9], or CADMOULD [10]. However, for example, the material database of the highly valuable Ansys-Polyflow [11] lacks such data.

Rheological data are typically limited to the Melt Flow Index (MFI), Melt Mass Flow Rate (MFR), or viscosity graphs. The parameters of the relevant rheological equations, necessary for full material characterization, are rarely available. Online rheological data are generally not available.

Due to the dynamic development of materials engineering and the continuous production of new polymeric materials, such as polymer blends, polymer composites (including wood plastic composites), and filled plastics, the situation becomes even more complicated. Material data for such materials are simply lacking in material databases, e.g., [7,8,9,10,11]. Such data are also lacking in the available literature on the subject, e.g., [12,13,14,15].

Well-described rheological properties of a material constitute the basis for process modeling, which is widely used in engineering practice. The lack of rheological data for advanced polymeric materials is a serious limitation of this modeling and its practical application in industry [16,17,18,19]. For example, this is indicated by the authors’ previous studies on modeling the extrusion of polymer blends [20,21,22,23,24,25,26,27] or wood plastic composites [28,29,30,31,32,33,34], as well as on modeling the injection molding [35,36].

Therefore, fast, effective, and inexpensive methods for determining the rheological properties of materials are essential. Effective methods for determining the parameters of rheological equations are also needed, which is especially important in the case of new materials that require time-consuming research.

This paper addresses the problem of determining viscosity curves for one of the most widely used advanced polymeric materials, namely, wood plastic composites. The studies were conducted in laboratory and production settings, i.e., on-line. The studies were conducted within a range of shear rates and temperatures, corresponding to typical processing conditions.

Laboratory tests were conducted in two ways, on the basis of classical rheometric measurements [37,38], i.e., High-Pressure Capillary Rheometry (HPCR), and on the basis of Melt Flow Index (MFI) measurements, also including tests based on a limited number of measurement points. These tests were described in three ways: by using the universal Vinogradov–Malkin equation, the two-parameter Ostwald–de Waele power law equation, and the reduced three-parameter Klein logarithmic equation.

Tests in production conditions, i.e., on-line, were carried out in the extrusion process based on the measurement of process output (material flow rate) and pressure measurement in a specialized extrusion die.

The test results (viscosity curves) obtained from Melt Flow Index (MFI) measurements and on-line measurements were presented and evaluated against the background of the results obtained from classical capillary rheometry measurements (HPCR).

The results of this part of the study (viscosity curves and models) will be used in the second part of the study to evaluate the impact of rheological testing methods and rheological models on the accuracy of process modeling (extrusion). Theoretical and experimental assessment will be conducted based on the analysis of polymer single-screw extrusion (wood plastic composites) under conditions of classic gravity feed (flood fed extrusion) and non-standard feed with dosing (starve fed extrusion), under various processing conditions.

## 2. WPCs Characteristics

Wood plastic composites are widely used in the economy, successfully replacing wood. They are resistant to atmospheric conditions, especially moisture. Composites based on polypropylene (PP), high-density polyethylene (HDPE), and polyvinyl chloride (PVC) are particularly important [13,14,15,39,40,41,42,43].

The level of knowledge in the field of processing and rheology of wood plastic composites is relatively poor. The basic sources in this field are the monographs by Mohanty et al. [13], Klyosov [14], and Oksman and Sain [15]; as well as the review papers by Li and Wolcott, e.g., [44,45,46], Xiao and Tzoganakis, e.g., [47,48,49,50], and the papers by Vlachopoulos and Hristov, e.g., [51,52,53]. Recently, valuable review contributions were presented by Farul et al. [54], Chan et al. [55], Khan et al. [56], Yadav et al. [57], Ramesh et al. [58], Elsheikh et al. [59], Sun et al. [60], and Mital’ová et al. [61].

Wood plastic composites are non-Newtonian, pseudoplastic materials. Their viscosity decreases with increasing shear rate and temperature, but increases with increasing wood flour content. Wood plastic composites can exhibit yield stress during flow and slippage on the walls of the flow channel. The slip rate increases with the shear rate, which can lead to plug flow. A higher percentage of wood flour in the composite also favors plug flow. A comprehensive review of knowledge regarding the rheology and processing of polymer wood composites is presented in [31,34,43].

## 3. Rheological Modeling

### 3.1. Laboratory Rheometry

#### 3.1.1. High-Pressure Capillary Rheometry (HPCR)

Capillary rheometry is the basis of rheological research in polymer processing. It involves determining the relationship between the volume flow rate of a material and the pressure gradient during pressure flow in a cylindrical channel (capillary). This flow is defined by the Hagen–Poiseuille equation:

(1) $$Q=\frac{\pi R^{4}}{8\mu }\frac{∆p}{L}$$

where Q is the volume flow rate, ∆p is the pressure gradient along the capillary length, R is the radius, L is the capillary length, and μ is the Newtonian viscosity.

On the basis of this equation, the viscosity can be determined according to the formula

(2) $$\mu =\frac{\pi R^{4}}{8Q}\frac{∆p}{L}$$

This formula can be transformed into the form

(3) $$\mu =\frac{\frac{∆pR}{2L}}{\frac{4Q}{\pi R^{3}}}$$

where the numerator expresses the shear stress $\tau_{w}$ on the channel wall, while the denominator expresses the shear rate ${\dot{\gamma }}_{w}$ on the wall.

Thus,

(4) $$\tau_{w}=\frac{∆pR}{2L}$$

(5) $${\dot{\gamma }}_{w}=\frac{4Q}{\pi R^{3}}$$

- Rabinowitsch–Mooney Correction

The Hagen–Poiseuille equation enables the determination of Newtonian viscosity, which is independent of shear rate. For non-Newtonian fluids, viscosity is a function of the shear rate.

The shear stress at the capillary wall is independent of the material constitutive equation and is expressed in the case of non-Newtonian fluids by the same Formula (4) as in the case of Newtonian fluids. However, the shear rate at the channel wall in the case of a non-Newtonian fluid is not equal to the shear rate of the Newtonian fluid and can not be expressed by Formula (5).

The Rabinowitsch–Mooney equation enables the determination of the shear rate in non-Newtonian flow, e.g., in the form

(6) $${\dot{\gamma }}_{w}=\frac{4Q}{\pi R^{3}}\left(\frac{3}{4}+\frac{1}{4}\frac{dln(4Q/\pi R^{3}}{dln\tau_{w}}\right)$$

The term 4Q/πR^3^ of this equation expresses, in accordance with Equation (5), the shear rate of the Newtonian fluid on the capillary wall. In the analysis of the flow of non-Newtonian fluids, this shear rate is called the nominal or uncorrected (or apparent) shear rate and is denoted by the symbol ${\dot{\gamma }}_{a}$, i.e.,

(7) $${\dot{\gamma }}_{a}=\frac{4Q}{\pi R^{3}}$$

Thus, Equation (6) can be written as

(8) $${\dot{\gamma }}_{w}={\dot{\gamma }}_{a}\left(\frac{3}{4}+\frac{1}{4}\frac{dln{\gamma ˙}_{a}}{dln\tau_{w}}\right)$$

where the derivative is the flow exponent, defined as

(9) $$\frac{dln\tau_{w}}{dln{\gamma ˙}_{a}}=n$$

and then the relationship is obtained:

(10) $${\dot{\gamma }}_{w}={\dot{\gamma }}_{a}\left(\frac{3n+1}{4n}\right)$$

In the case of shear-thinning fluids, also known as pseudoplastic fluids, i.e., those for which viscosity decreases with increasing shear rate, the flow exponent n is less than unity, i.e., n < 1.

In the case of shear-thickening fluids, also known as dilatant fluids, i.e., those for which viscosity increases with increasing shear rate, the flow exponent n is greater than unity, i.e., n > 1.

In the case of Newtonian fluids, i.e., those for which viscosity does not depend on shear rate, the flow exponent n is equal to unity, i.e., n = 1.

The shear rate ${\dot{\gamma }}_{w}$ in a non-Newtonian fluid at the capillary wall is called the corrected shear rate or true shear rate. The expression in the parentheses of Equation (10) can be thought of as a correction to the nominal shear rate ${\dot{\gamma }}_{a}$, and this correction is called the Rabinowitsch–Mooney correction (shortly Rabinowitsch correction).

- Bagley Correction

The capillary flow analysis presented above is based on the assumption that the flow is fully developed along the entire capillary length, and the pressure drop is solely the result of internal fluid friction. This ignores the existence of end-flow effects at the capillary inlet and outlet, which result in additional pressure drops in the capillary, with the inlet effects being particularly important. As a result of this neglect, the calculations of the shear stress, defined by Formula (4), are subject to some error and provide values higher than would be expected based on the adopted assumptions.

The inlet effect is related to the formation of a velocity profile in the initial part of the capillary. Additional energy is required for this formation, resulting in a higher pressure gradient in this region than in the fully formed flow region. This raises the problem of determining, based on experimental data, the value of the pressure drop caused by internal friction in the fully formed velocity profile. The measurements result in a combined pressure drop resulting from the inlet effect and the formed flow, i.e.,

(11) $$∆p_{c}=∆p+∆p_{e}$$

where $∆p_{c}$ is the actual (i.e., measured during rheometric measurements) pressure drop, ∆p is the theoretical pressure drop ∆p, occurring as a result of flow in the capillary (under the conditions of the fully formed velocity profile), and $∆p_{c}$ is the additional pressure drop at the capillary inlet (inlet loss).

The Bagley method of eliminating the inlet effect consists of relating the actual pressure drop $∆p_{c}$ to a certain hypothetical capillary length L_c_, which also includes the additional pressure drop in the inlet section L_e_.

So there is a connection here:

(12) $$\frac{∆p}{L}=\frac{∆p_{c}}{L_{c}}$$

in which L_c_ = L + ∆L, and ∆L = eR is the additional (fictitious) capillary length, taking into account the additional pressure drop in the inlet section L_e_; e is the correction factor, the so-called Bagley correction; and R is the capillary radius.

The shear stress defined by Formula (4) can now be calculated from the relationship

(13) $$\tau_{w}=\frac{∆p_{c}R}{2(L+eR)}$$

The value of the Bagley correction factor can be determined based on measurements made for capillaries with different L/R ratios. For each capillary, the pressure drop ∆p_c_ should be determined, giving a certain constant value of the uncorrected shear rate ${\dot{\gamma }}_{a}$, defined by Formula (7). The graph of the dependence (14)

(14) $$∆p_{c}=f\left(\frac{L}{R}\right)$$

should be a straight line, cutting off the value of e on the L/R abscissa axis for ∆p_c_ = 0. Similar graphs can be made for other constant values of the shear rate ${\dot{\gamma }}_{a}$, thus obtaining the relationship

(15) $$e=f({\dot{\gamma }}_{a})$$

which enables the calculation of the shear stress $\tau_{w}$ according to Formula (13), for any value of the shear rate ${\dot{\gamma }}_{a}$.

It is worth noting that the influence of inlet losses on the measurement results decreases with the ratio of the capillary length L to the diameter D. This influence is usually considered negligible when L/D > 60.

- Mooney Correction

Capillary flow analysis is typically conducted assuming no slippage on the capillary wall, meaning the fluid velocity on the wall is assumed to be equal to the wall velocity, i.e., zero. In reality, such slippage often occurs in the flow of polymeric materials. It occurs when a critical shear stress on the capillary wall is exceeded.

Slippage is an important phenomenon, but difficult to assess. The slippage velocity can be determined using the Mooney method [4,62]. To illustrate the essence of this method, consider the flow without slippage and the flow with slippage. In the former case, the flow rate Q in a capillary of radius R will be equal:

(16) $$Q=\pi R^{2}V$$

where V is the mean flow velocity.

The shear rate ${\dot{\gamma }}_{a}$ will be expressed by Formula (7), which, after applying Formula (16), will take the form

(17) $${\dot{\gamma }}_{a}=\frac{4V}{R}$$

In the case of slippage, the shear rate will be given by

(18) $${\dot{\gamma }}_{as}=\frac{4\left(V-V_{s}\right)}{R}$$

where (V − V_s_) is the mean flow velocity of the material relative to its velocity on the capillary wall.

After introducing the relationship (7), we obtain

(19) $${\dot{\gamma }}_{as}=\frac{4Q}{\pi R^{3}}-\frac{4V_{s}}{R}$$

The Mooney method involves taking a series of measurements of the uncorrected shear rate ${\dot{\gamma }}_{a}$ for capillaries of different radii but with the same L/R ratio. This is performed while maintaining a constant value of the shear stress $\tau_{w}$, i.e., at a constant pressure drop ∆p. Then, the obtained results are expressed as a function of the inverse of the capillary radius 1/R. A linear relationship is then obtained, which corresponds to Equation (19), transformed to the form

(20) $$\frac{4Q}{\pi R^{3}}={\dot{\gamma }}_{as}+\frac{4V_{s}}{R}$$

The slope of the obtained line is equal to 4 V_s_, so it determines the value of the slip velocity. Extrapolation of the obtained relationship for R → ∞, i.e., for 1/(R = 0), determines the shear rate ${\dot{\gamma }}_{as}$, taking into account the occurrence of slip.

- Analysis of Rheological Corrections

**Figure 1:**
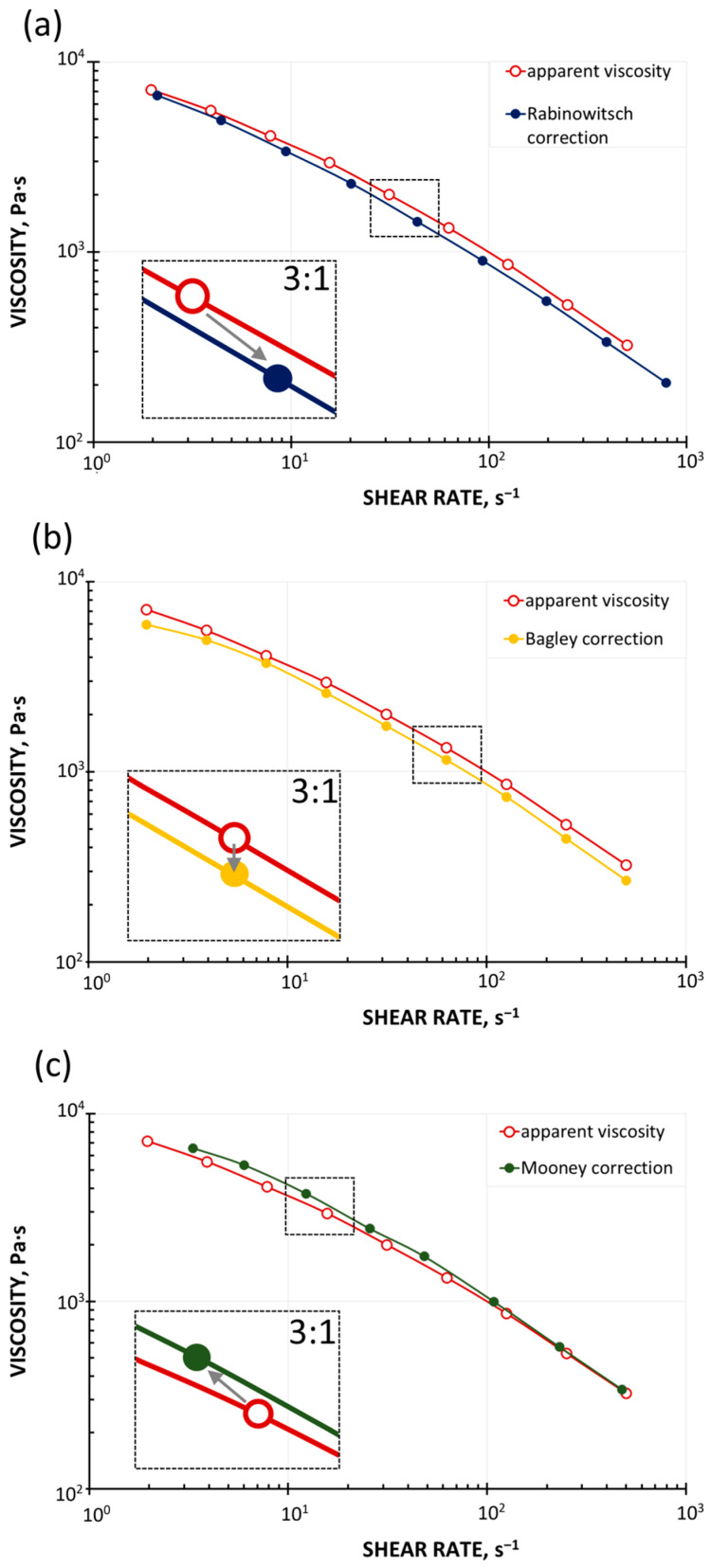
Effect of rheometric corrections on viscosity curves: (**a**) Rabinowitsch correction, (**b**) Bagley correction, and (**c**) Mooney correction [34].

It is worth presenting the effect of the Rabinowitsch, Bagley, and Mooney corrections on the position of the viscosity curve relative to the uncorrected curve. There is no such discussion in the literature, except [34]. The basis for this discussion is Figure 1.

The introduction of the Rabinowitsch correction, defined by Equation (10), means a change in the shear rate at constant shear stress (the calculated pressure gradient in the capillary does not change). In the case of shear-thinning (pseudoplastic) fluids, for which n < 1, the shear rate at the capillary wall increases, causing the viscosity to take on lower values. The viscosity curve therefore shifts downward, toward higher shear rates, i.e., diagonally to the right (Figure 1a). In the case of shear-thickening (dilatant) fluids, for which n > 1, the shear rate at the capillary wall decreases, causing the viscosity to take on higher values. The viscosity curve therefore shifts upward, toward lower shear rates, i.e., diagonally to the left (Figure 1a).

The introduction of the Bagley correction, defined by Equation (13), reduces the shear stress due to the inlet pressure losses at a constant shear rate, which causes the viscosity to take lower values. The viscosity curve therefore shifts downward, but vertically, since the shear rate does not change (Figure 1b).

The introduction of the Mooney correction, defined by Equation (19), reduces the shear rate at constant shear stress (the calculated pressure gradient in the capillary remains constant), which causes the viscosity to assume higher values. The viscosity curve therefore shifts upward, toward lower shear rates, i.e., diagonally to the left (Figure 1c).

Of course, the final position of the viscosity curve depends on the type of material being tested, as well as the measurement conditions, temperature, and shear rate range.

#### 3.1.2. MFI (Melt Flow Index) Rheometry

Viscosity curves of polymeric materials are typically determined from multiple measurement points over a wide range of shear rates and temperatures using capillary rheometers. The results of these studies are then approximated by rheological models describing the dependence of viscosity on shear rate and temperature, e.g., by the Bird–Carreau, Klein, or Cross-WLF models, which are used, among others, in computer simulations of processing, e.g., [4,63].

The problem of tedious determination of viscosity curves within a selected range of shear rates and temperatures can be addressed to some extent by using limited measurements, e.g., based on one, two, or three measurement points obtained by measuring the Melt Flow Index (MFI) using a plastometer (Melt Indexer). This paper proposes solutions based on the universal Vinogradov–Malkin equation (for one measurement point), the Ostwald–de Waele equation (for two measurement points), and the reduced Klein equation (for three measurement points).

Viscosity is defined by the ratio of shear stress to shear rate:

(21) $$\eta =\frac{\tau }{\dot{\gamma }}$$

where η is the viscosity, τ is the shear stress, and $\dot{\gamma }$ is the shear rate.

The shear stress on the capillary wall depends on the load applied during measurement, and is expressed as

(22) $$\tau_{w}=\frac{∆pR_{cap}}{2L_{cap}}$$

where

(23) $$∆p=\frac{Mg}{A}$$

where $\tau_{w}$ is the shear stress (on the capillary wall), ∆p is the pressure gradient along the capillary length, R_cap_ is the capillary radius, M is the piston load, g is the acceleration of gravity, and A is the cross-sectional area of the piston.

Thus, for a standardized geometry of the measuring device (capillary, piston, and cylinder), the shear stress (on the capillary wall) can be represented as

(24) $$\tau_{w}=\frac{MgR_{cap}}{2L_{cap}A}\;$$

The shear rate under pressure flow conditions in a cylindrical channel (capillary) is expressed as

(25) $${\dot{\gamma }}_{w}=\frac{4Q}{\pi R_{cap}^{\;\;\;\;\;\;\;3}}\;$$

where ${\dot{\gamma }}_{w}$ is the shear rate (on the capillary wall), Q is the volume flow rate, and R_cap_ is the capillary radius.

It is worth noting here that the Melt Flow Index (MFI) defines the mass flow rate in g/10 min, from which the volume flow rate can be obtained, expressed as

(26) $$Q=\frac{MFI}{600\rho_{m}}\;\;$$

where Q is the volume flow rate, cm^3^/s; MFI is the value of Melt Flow Index, g/10 min; and ρ_m_ is the polymer melt density, g/cm^3^.

Thus, dependencies were obtained that enable the determination of viscosity based on the Melt Flow Index (MFI), the plastometer piston load M, and the material density ρ_m_.

The viscosity values obtained in this way, determined for different shear rates, can be described by an appropriate rheological equation, which makes it possible to determine the full viscosity curve of the material. The accuracy of such measurements is subject to a systematic error that increases the viscosity value due to the use of a short capillary and the omission of the Bagley correction. However, the obtained results are often close to those obtained with a capillary rheometer, and the achievable shear rate range (from a few 1/s to approximately 1000 1/s) adequately covers the shear rate range found in polymer processing. The existence of a systematic error is confirmed by information from Goettfert [38].

- One-Point Method of Determining the Viscosity Curve—Vinogradov–Malkin Model

Determination of the viscosity curve is possible based on a one-point measurement using the so-called universal viscosity curve, defined by the Vinogradov–Malkin model [64,65,66], who showed that for most thermoplastic polymers (without fillers), the following relationship is valid:

(27) $$n\;=\;\eta_{0}/1+A_{1}\cdot {\left(\dot{\gamma }\cdot \eta_{0}\right)}^{\alpha }+\;A_{2}\cdot {\left(\dot{\gamma }\cdot \eta_{0}\right)}^{2\alpha }$$

where *η* is the viscosity at shear rate $\dot{\gamma }$ at temperature T; *η*_0_ is the zero viscosity at zero shear rate ($\dot{\gamma }$ → 0) at temperature T; $\dot{\gamma }$ is the shear rate at a given measuring point; A_1_, A_2_, and α are the universal coefficients of the equation, A_1_ = 1.386 × 10^−2^; A_2_ = 1.462 × 10^−3^; α = 0.355.

Based on a single measurement of the viscosity η, e.g., by measuring the Melt Flow Index (MFI) and the corresponding shear rate $\dot{\gamma }$, the zero viscosity η_0_ can be determined from Equation (27) in an iterative procedure.

- Two-Point Method of Determining the Viscosity Curve—Ostwald–de Waele Model

The two-point method proposed in this work [3,4] should be more accurate, consisting of linear approximation (in a double-logarithmic system) of two measurement points, based on the Ostwald–de Waele power law equation, which has the form

(28) $$\tau =k\;{\dot{\gamma }}^{n}$$

where k is the consistency coefficient, and n is the flow exponent.

To determine the consistency k and the flow exponent n, two Melt Flow Index measurements should be performed, one for a Low Load (e.g., 1.2 kg), i.e., low shear rate, and one for a High Load (e.g., 15 kg), i.e., a high shear rate.

Then, Equation (8) for the Low Load can be written as

(29) $$\tau_{\mathrm{LL}}=k\;{\dot{\gamma }}_{LL}^{n}$$

and for High Load as

(30) $$\tau_{\mathrm{HL}}=k\;{\dot{\gamma }}_{HL}^{n}$$

After taking the logarithm of both sides of these equations and transforming them into linear form, and then subtracting both sides, we obtain the relationship

(31) $$\mathrm{lg}\tau_{\mathrm{LL}}-\mathrm{lg}\tau_{\mathrm{HL}}=n(\mathrm{lg}{\dot{\gamma }}_{LL}^{n}-{\dot{\gamma }}_{HL}^{n})$$

from which the flow exponent n can be determined as

(32) $$n=\frac{lg\tau_{HL}-lg\tau_{LL}}{lg{\dot{\gamma }}_{HL}-lg{\dot{\gamma }}_{LL}}$$

The shear stress on the capillary wall can be written as

(33) $$\tau_{w}=\frac{∆pR_{cap}}{2L_{cap}}=\frac{\frac{Mg}{\pi R_{c}^{\;\;2}}R_{cap}}{2L_{cap}}=M\frac{\frac{gR_{cap}}{\pi R_{c}^{2}}}{2L_{cap}}$$

where

(34) $$\frac{\frac{gR_{cap}}{\pi R_{c}^{2}}}{2L_{cap}}=const=\alpha$$

The shear rate can in turn be written as

(35) $$\dot{\gamma }=\frac{4Q}{\pi R_{cap}^{\;\;\;\;\;\;3}}=\frac{4\frac{MFI}{\rho_{m}}}{\pi R_{cap}^{\;\;\;\;\;\;\;3}}=MFI\frac{4}{\rho_{m}\pi R_{cap}^{3}}$$

where

(36) $$\frac{4}{\rho_{m}\pi R_{cap}^{\;\;\;\;\;\;\;3}}=const=\beta$$

As a result, the flow exponent n can be represented as

(37) $$n=\frac{\mathrm{lg}\left(\alpha M_{HL}\right)-lg(\alpha M_{LL})}{\mathrm{lg}\left(\beta {MFI}_{HL}\right)-lg(\beta {MFI}_{LL})}$$

The consistency coefficient k can be expressed as

(38) $$k=\frac{\tau_{LL}}{{\dot{\gamma }}_{LL}^{n}}=\frac{\alpha M_{LL}}{{\left(\beta {MFI}_{LL}\right)}^{n}}$$

Knowing the parameters of the power law equation, it is now possible to determine the viscosity of the material for any shear rate.

- Three-Point Method of Determining the Viscosity Curve—Klein Model

In order to increase the accuracy of approximation, a three-point method can be proposed, which involves determining the viscosity for three, as equally spaced as possible (on a logarithmic scale), Melt Flow Index measurement points.

The viscosity measurement results can then be approximated by the appropriately reduced Klein equation [67], from which the temperature influence term is removed:

(39) $$ln\eta \;+\;A_{0}+A_{1}\ln\dot{\gamma }+A_{11}\ln^{2}\dot{\gamma }$$

This Equation (39) can be reduced to a quadratic equation as

(40) $$y\;=\;{ax}^{2}\;+\;bx\;+\;c$$

where y = ln*η*, x = ln$\dot{\gamma }$, a = A_11_, b = A_1_, and c = A_0_.

### 3.2. On-Line Rheometry

The rheological characteristics of the tested material can be determined in the extrusion process based on the measurement of the process output (material flow rate) and pressure measurement in a specialized extrusion die.

**Figure 2:**
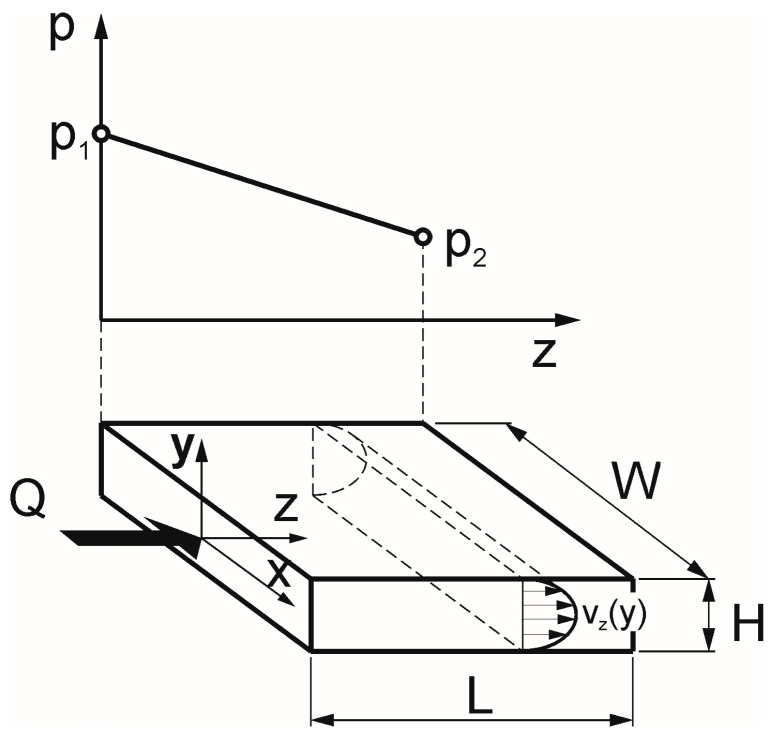
The flow in the flat channel (between two parallel plates): Q—volume flow rate, p—pressure, p_1_—pressure at channel entrance, p_2_—pressure at channel exit, L, W, H—channel dimensions, and x, y, z—coordinates.

**Figure 3:**
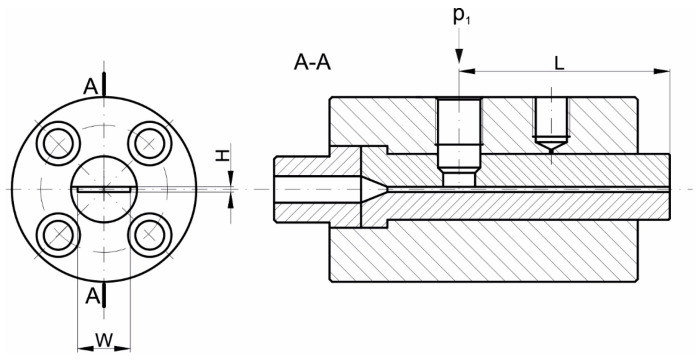
Geometric scheme of a flat (slit) measuring mouthpiece: p_1_—pressure measurement point, L—measuring length, W—channel width, and H—channel height.

The basis of the calculation procedure is the analysis of the flow in the flat channel (between two parallel plates), which is schematically shown in Figure 2. A geometric scheme of a flat (slit) measuring mouthpiece is depicted in Figure 3.

Viscosity is defined as

(41) $$\;\eta =\frac{\tau_{w}}{{\dot{\gamma }}_{w}}$$

where η is the viscosity, $\tau_{w}$ is the shear stress at the channel wall, and ${\gamma ˙}_{w}$ is the shear rate at the channel wall.

The shear stress at the wall in the flat channel is expressed as follows (Figure 2):

(42) $$\tau_{yz}\left(y=H/2\right)=-\frac{∆pH}{2L}$$

where ∆p is the pressure difference, ∆p = p_1_ − p_2_, p_1_ is the measured pressure, p_2_ is the atmospheric pressure, H is the channel height, and L is the channel length.

The shear rate at the wall in the flat channel is expressed as

(43) $${\dot{\gamma }}_{w}=\frac{dv_{z}}{dy}\left(y=H/2\right)=\frac{6Q}{WH^{2}}$$

where Q is the volume flow rate, W is the channel width, and H is the channel height.

An effect of the side walls of the channel on the relationship between a flow rate and a pressure gradient can be expressed by the shape coefficient [68,69] as follows:

(44) $$∆p=\frac{12\eta QL}{WH^{3}}\frac{1}{f_{p}}$$

where ∆p is the pressure difference; Q is the volume flow rate, η is the viscosity; W, H, L are the channel dimensions; amd f_p_ = f_p_ (H/W) is the shape coefficient.

The power law model was used for a rheological description of the composite under study. This model is expressed as

(45) $$\tau =k{\dot{\gamma }}^{n}$$

where k is the consistency coefficient, Pa∙s^n^, and n is the flow exponent (dimensionless).

Using this model, the viscosity can be expressed as

(46) $$\eta =k{\dot{\gamma }}^{n-1}$$

and after logarithmization

(47) $$lg\;\eta =\mathrm{lg}k+\left(n-1\right)\mathrm{lg}\dot{\gamma }$$

Using linear regression, the parameters of the power law model, the consistency coefficient k and the flow exponent n, can be calculated from this equation.

The viscosity dependence on the temperature can be expressed as

(48) $$\eta \left(T\right)=\eta_{r}exp\left[-b\left(T-T_{r}\right)\right]$$

where η(T) is the viscosity at the temperature T, η_r_ is the reference viscosity at the reference temperature T_r_, b is the material constant, 1/°C, T is the temperature, and T_r_ is the reference temperature.

The material constant b can be expressed and calculated as

(49) $$b=-\frac{lg\;\eta \left(T\right)-lg\;\eta_{r}}{T-T_{r}}$$

Using Equation (49), you can determine the parameter b when you know the viscosity η at two different temperatures, T and T_r_.

## 4. Material

The wood polymer composite (PP copo inj 4, Beologic, Sint-Denijs, Belgium) composed of polypropylene (PP) matrix and 25% wood filler was applied in this research. The polypropylene density was equal to ρ_PP_ = 0.9 g/cm^3^, the polymer Melt Flow Index was equal to MFI = 25 g/10 min (at M = 2.16 kg, and T = 230 °C), and the melting temperature was equal to T_m_ = 160 °C. The composite solid density was equal to ρ_s_ = 1.2 g/cm^3^, the melt density ρ_m_ = 0.85 g/cm^3^, the composite bulk density ρ_b_ = 0.4–0.6 g/cm^3^, and the polymer Melt Flow Index was equal to MFI = 8.3 g/10 min (at M = 2.16 kg, and T = 190 °C). More detailed material data were unavailable, which limits the ability to accurately repeat the measurement results.

## 5. Experimental

### 5.1. Laboratory Measurements

Laboratory tests were conducted in two ways, on the basis of classical rheometric measurements, i.e., High-Pressure Capillary Rheometry (HPCR) and on the basis of Melt Flow Index (MFI) measurements, also including tests based on a limited number of measurement points.

#### 5.1.1. Determination of Viscosity Based on High-Pressure Capillary Rheometry (HPCR)

Capillary rheometry is the basis of rheological research in polymer processing. In this paper, the results of capillary rheometry tests (viscosity curves) will be the reference viscosity curves for the test results (viscosity curves) obtained from Melt Flow Index measurements and on-line measurements.

The WPC viscous properties were measured at various temperatures with the use of a capillary rheometer (RG-25, Goettfert, Buchen, Germany). The capillaries of diameter D = 1 mm and the length/diameter ratio L/D = 0.2/1, 10/1 were used, and the Rabinowitsch correction and the Bagley correction were applied (according to Goettfert’s information, the length of the capillary L/D = 0.2/1 is the lower limit for acceptable rheological practice). The measurements were carried out for the temperatures T = 180 °C, 185 °C, and 190 °C, and for the shear rates from $\dot{\gamma }$ = 10 s^−1^ to $\dot{\gamma }$ = 5000 s^−1^.

**Figure 4:**
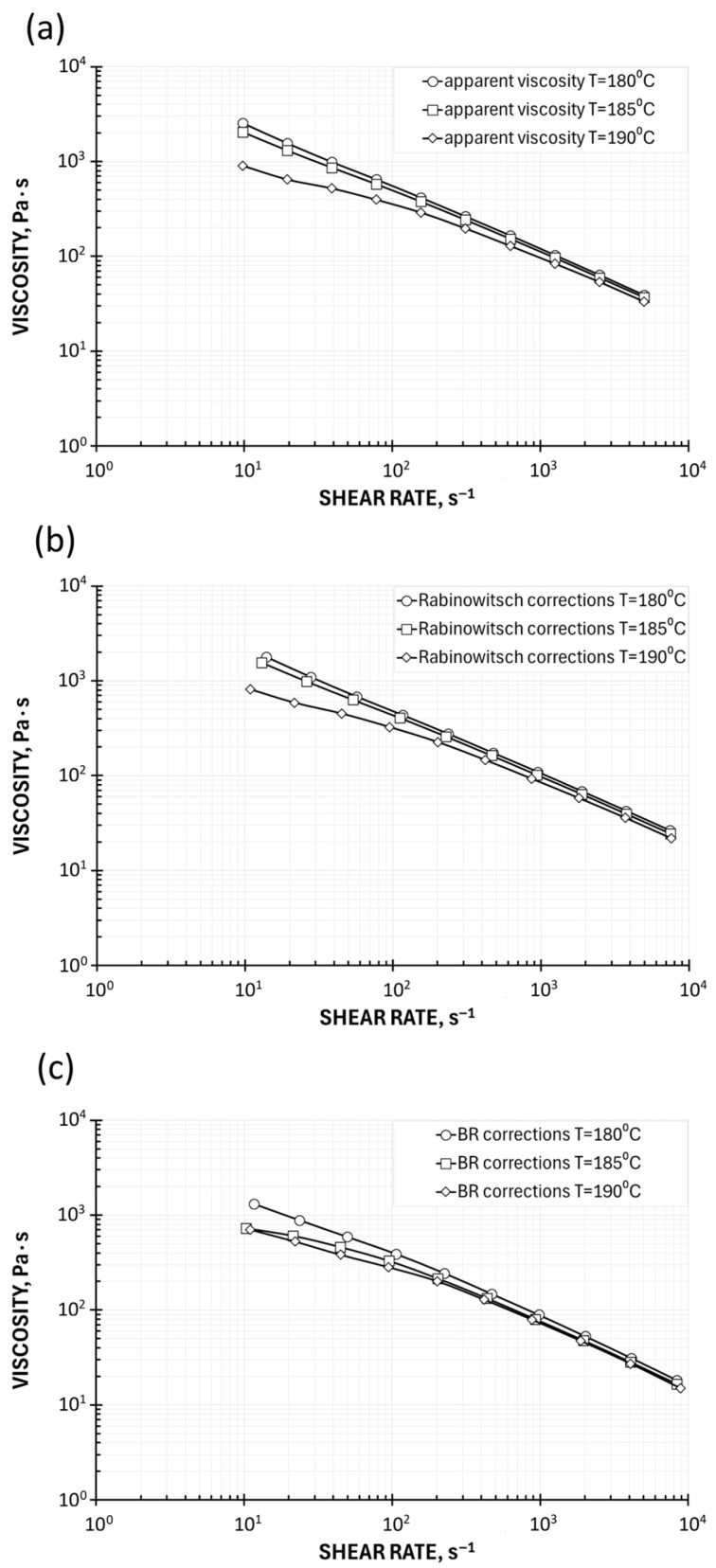
Viscosity curves determined using High-Pressure Capillary Rheometry: (**a**) apparent viscosity, (**b**) Rabinowitsch correction, and (**c**) Rabinowitsch and Bagley corrections.

The viscosity characteristics at the temperatures T = 180 °C, 185 °C, and 190 °C are shown in Figure 4. An obvious pseudoplastic behavior is observed, indicating that the viscosity decreases with increasing shear rate. The viscosity also decreases as the temperature increases, although the effect of temperature on viscosity is not high.

An effect of rheometric corrections is also clearly seen. Introduction of the Rabinowitsch correction, defined by Equation (10), means a change in the shear rate at constant shear stress (the calculated pressure gradient in the capillary does not change). In the case of shear-thinning (pseudoplastic) fluids, for which n < 1, the shear rate at the capillary wall increases, causing the viscosity to take on lower values. The viscosity curve therefore shifts downward, toward higher shear rates, i.e., diagonally to the right (Figure 4b). Introduction of the Bagley correction, defined by Equation (13), reduces the shear stress due to the inlet pressure losses at a constant shear rate, which causes the viscosity to take lower values. The viscosity curve therefore shifts downward, but vertically, since the shear rate does not change (Figure 4c).

It is worth noting that the conducted studies did not address the issue of potential slippage on the flow channel walls and yield stress, as these are only possible with capillary rheometry measurements, and plastometric measurements do not provide this capability. Slippage and yield stress can lead to plug flow. In such cases, rheometric calculations must be modified accordingly, using, for example, Mooney analysis [4,62], described by Equations (16)–(20).

It is worth noting that the viscosity of composites can be evaluated based on the viscosity of the matrix and the filler content, using the well-known equations of Einstein/Batchelor or Krieger/Dougherty, as demonstrated by Le Moigne et al. [70] and Polychronopoulos et al. [71].

#### 5.1.2. Determination of Viscosity Based on Melt Flow Index (MFI)

Viscosity can be determined from Melt Flow Index (MFI) measurements according to the procedure described by Equations (21)–(26). Furthermore, according to this procedure, viscosity curves can be determined based on measurements made at different piston loads.

The measurements were taken using a Melt Indexer (MI-2, Goettfert, Buchen, Germany), operating on the principle of constant shear stress. The viscosity curves were determined based on seven measurement points resulting from the applied load (1.2, 2.16, 3.8, 5.0, 10.0, 12.5, and 15.0 kg). A standardized capillary with a diameter of D_cap_ = 2.095 mm, a length of L_cap_ = 8.0 mm, and a L_cap_/D_cap_ ratio of 3.819 was used. The cylinder diameter was equal to D_c_ = 9.55 mm. The measurements were carried out for the temperatures T = 180 °C, 185 °C, and 190 °C, and for the shear rates from $\dot{\gamma }$ = 10 s^−1^ to $\dot{\gamma }$ = 1000 s^−1^.

**Figure 5:**
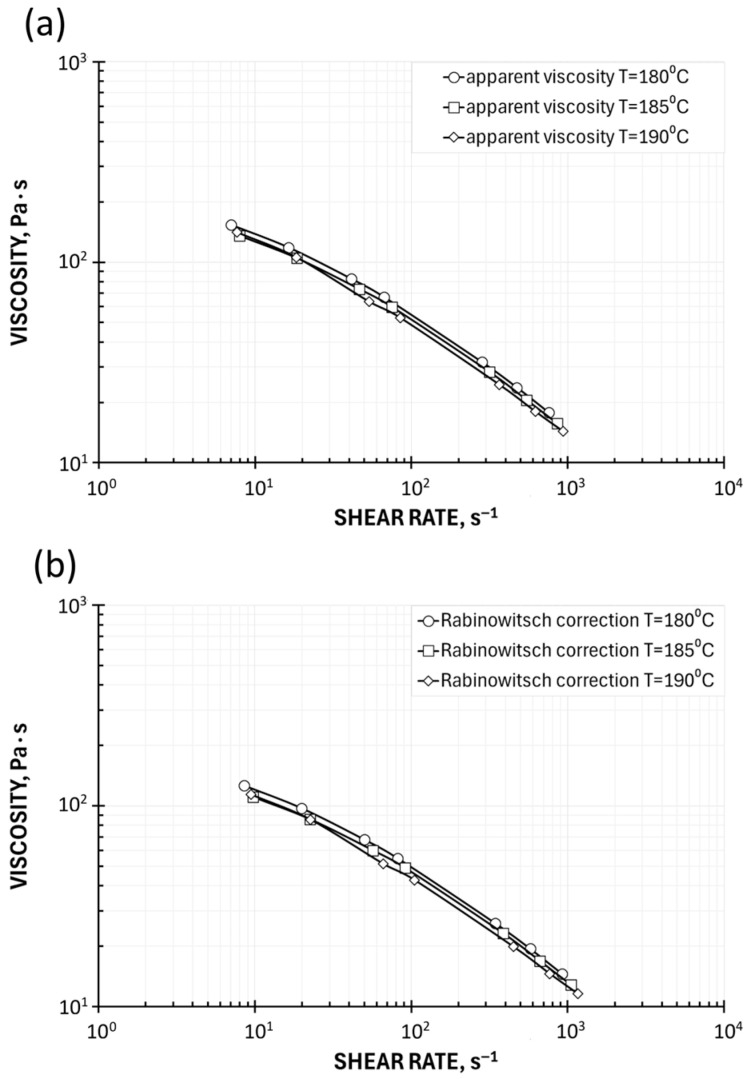
Viscosity curves determined using Melt Flow Index: (**a**) apparent viscosity and (**b**) Rabinowitsch correction.

The viscosity characteristics at the temperatures T = 180 °C, 185 °C, and 190 °C are shown in Figure 5. A pseudoplastic behavior is obviously seen, which means the viscosity decreases with an increase in the shear rate. The viscosity also decreases as the temperature increases. The effect of temperature on viscosity is not high.

An effect of Rabinowitsch correction is also clearly seen in High-Pressure Capillary Rheometry (HPCR). An introduction of this correction, defined by Equation (10), means a change in the shear rate, which increases at the capillary wall, causing the viscosity to take on lower values. The viscosity curve, therefore, shifts downward, toward higher shear rates, i.e., diagonally to the right (Figure 5b). It is worth noting that it is not possible to apply the Bagley correction using Melt Flow Index-based measurements.

**Figure 6:**
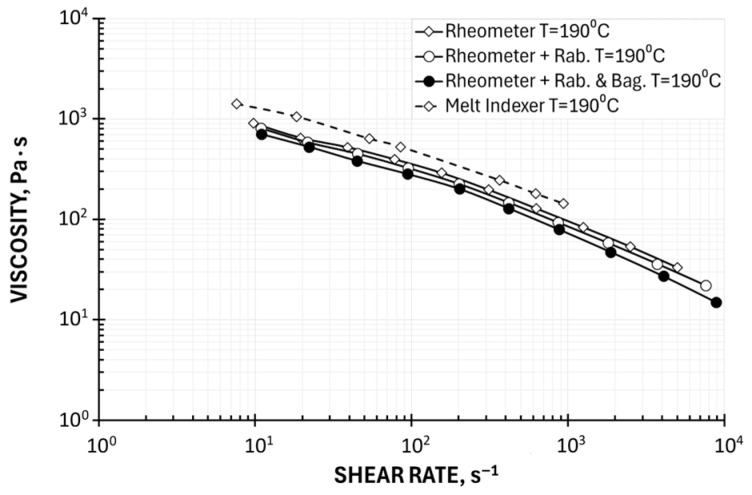
Viscosity curves determined using High-Pressure Capillary Rheometry (HPCR) and Melt Flow Index (MFI) measurements (apparent viscosity).

**Figure 7:**
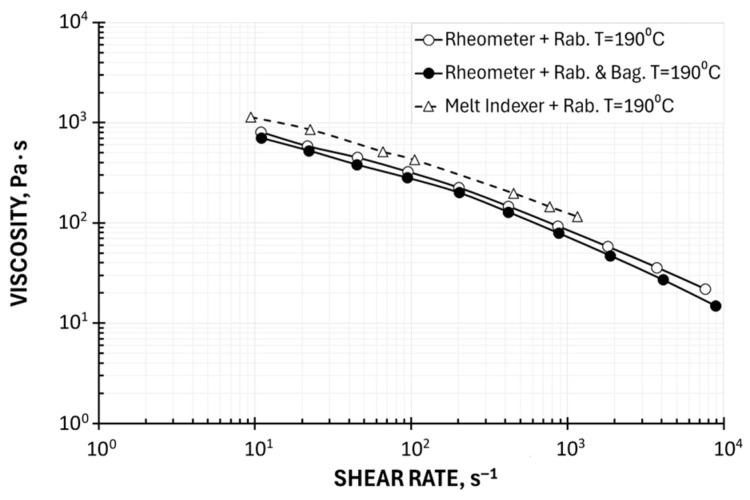
Viscosity curves determined using High-Pressure Capillary Rheometry (HPCR) and Melt Flow Index (MFI) measurements (with Rabinowitsch correction).

A comparison of rheometric (HPCR) and plastometric (MFI) measurement results is presented in Figure 6 and Figure 7. Figure 6 shows the results obtained without rheometric corrections, and Figure 7 shows the results obtained using the Rabinowitsch and Bagley corrections.

The accuracy of plastometric (MFI) measurements is subject to errors that increase the viscosity value, which results from the use of a short capillary and the omission of the Bagley correction and the failure to take into account the Rabinowitsch correction (Figure 6). It should be noted, however, that it is possible to apply the Rabinowitsch correction in plastometric studies, as was performed in this work (Figure 7). This reduces the discrepancies between the results of rheometric and plastometric measurements.

Despite significant discrepancies in the results of rheometric and plastometric tests, it should be noted that the obtained results of plastometric measurements reflect the nature of the dependence of viscosity on shear rate, and the achievable shear rate range (from $\dot{\gamma }$ = 10 s^−1^ to $\dot{\gamma }$ = 1000 s^−1^) with a typical set of loads largely covers the shear rate range occurring, for example, in the polymer extrusion process. The shift of the viscosity curves toward higher values compared to the rheometric studies confirms the information from Goettfert [38].

Plastometric studies across the full available shear rate range (based on multiple measurement points) are tedious and time-consuming, and the results obtained still differ from rheometric studies. Therefore, it is justified to seek research methods based on a limited number of measurement points.

In this paper, we propose three methods for determining viscosity based on a limited number of measurements. The test results (viscosity curves) obtained from Melt Flow Index measurements were described in three ways: by using the universal Vinogradov–Malkin equation, the two-parameter Ostwald–de Waele power law equation, and the reduced three-parameter Klein logarithmic equation.

- One Point Method of Determining the Viscosity Curve—Vinogradov–Malkin Model

Based on a single measurement of the viscosity η, e.g., by measuring the Melt Flow Index (MFI) and the corresponding shear rate $\dot{\gamma }$, the zero viscosity η_0_ can be determined from Equation (27) in an iterative procedure. In this paper, the Solver module of MS Excel was used for this purpose. As a result, the viscosity curve of the material at the temperature T was obtained.

**Figure 8:**
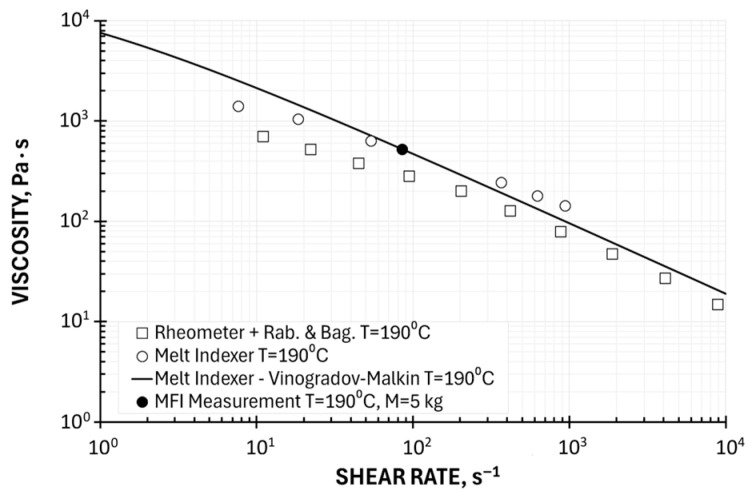
Viscosity curve determined using Vinogradov–Malkin model based on a one-point measurement, M = 5 kg, in relation to plastometric (MFI) measurements and rheometric (HPCR) measurements (Rabinowitsch and Bagley correction).

An example of computations, for the temperature T = 190 °C (the zero viscosity η_0_ = 27,453 Pa·s) is depicted in Figure 8. The viscosity curve determined using the Vinogradov–Malkin model based on a one-point measurement is shown in relation to plastometric (MFI) measurements and in relation to rheometric (HPCR) measurements (with Rabinowitsch and Bagley correction). This curve corresponds relatively well to the results of plastometric tests but differs significantly from the results of rheometric tests. It should also be noted that the form of the Vinogradov–Malkin equation depends on the choice of the measurement point. Furthermore, this equation has limited applications in analytical solutions to polymer processing issues.

An error in using this method depends on the type of material. It is impossible to describe the properties of different materials using a curve constructed from a single measurement point. It is possible, however, to tailor the Vinogradov–Malkin curve to the material being tested by properly selecting the A_1_, A_2_, and α parameters. However, this is a time-consuming process and requires more than one viscosity measurement point.

- Two Point Method of Determining the Viscosity Curve—Ostwald–de Waele Model

Determining the viscosity curve from a single measurement point using universal coefficients, without an additional geometric point to define this curve, is prone to significant error. The two-point method is more accurate, consisting of linear approximation (in a double-logarithmic system) of the results obtained at two measurement points using the Ostwald–de Waele power law model, defined by Equation (28). The procedure for determining viscosity curves is described by Equations (29)–(38).

In the paper, we applied this approach in two ways: in the full available shear rate range, i.e., at loads M_LL_ = 1.2 kg and M_HL_ = 15 kg, and in the shear rate range corresponding to the conditions of polymer processing (extrusion), i.e., at loads M_LL_ = 5 kg and M_HL_ = 15 kg.

**Figure 9:**
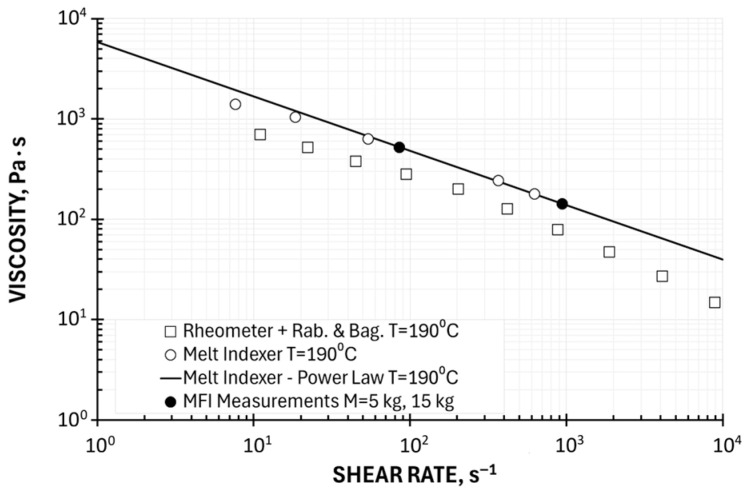
Viscosity curve determined using Ostwald–de Waele model based on a two-point measurement, M = 1.2 kg, 15 kg, in relation to plastometric (MFI) measurements and rheometric (HPCR) measurements (Rabinowitsch and Bagley correction).

**Figure 10:**
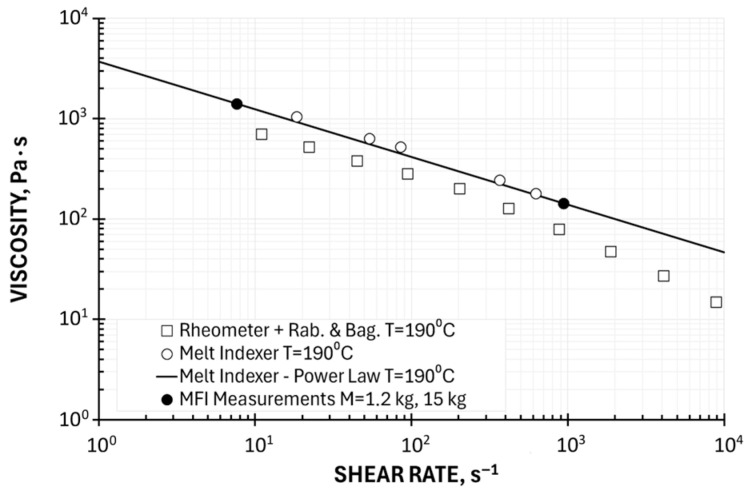
Viscosity curve determined using Ostwald–de Waele model based on a two-point measurement, M = 5 kg, 15 kg, in relation to plastometric (MFI) measurements and rheometric (HPCR) measurements (Rabinowitsch and Bagley correction).

Examples of computations for the temperature T = 190 °C (at M_LL_ = 1.2 kg, M_HL_ = 15 kg: the consistency coefficient k = 3698 Pa·s^n^, the flow exponent n = 0.525; at M_LL_ = 5 kg and M_HL_ = 15 kg: the consistency coefficient k = 5878 Pa·s^n^, the flow exponent n = 0.457) are depicted in Figure 9 and Figure 10. Viscosity curves determined using the Ostwald–de Waele model based on a two-point measurement are shown in relation to plastometric (MFI) measurements and in relation to rheometric (HPCR) measurements (with Rabinowitsch and Bagley correction). These curves correspond well to the results of plastometric tests but differ from the results of rheometric tests. It should also be noted that the parameters of the Ostwald–de Waele model depend on the choice of measurement points, i.e., the range of shear rates. A very important advantage of the power law model is the ability to solve polymer processing issues in an analytical way.

- Three Point Method of Determining the Viscosity Curve—the reduced Klein Model

Determining viscosity based on two measurement points is sufficient for basic engineering applications, especially within the shear rate range corresponding to the Melt Flow Index measurements. However, outside this range, a straight line (in the logarithmic system) does not describe the tested composite very well.

In order to increase the accuracy of approximation, a three-point method using the reduced Klein model (39) can be proposed, which involves determining the viscosity for three, as equally spaced as possible (on a logarithmic scale), Melt Flow Index measurement points. The parameters of the Klein equation (A_0_, A_1_, and A_11_) were determined, using rearrangement (40), via linear regression applying MS Excel, using the REGLINP statistical function in the form of tables.

**Figure 11:**
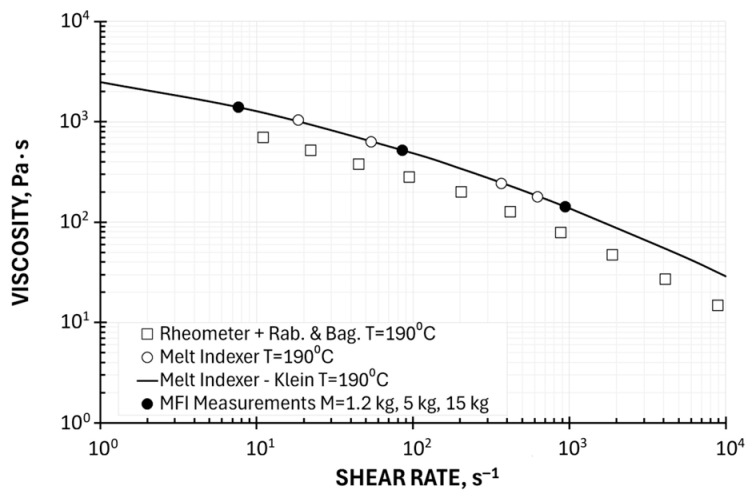
Viscosity curve determined using reduced Klein model based on a three-point measurement, M = 1.2 kg, 5 kg, 15 kg, in relation to plastometric (MFI) measurements and rheometric (HPCR) measurements (Rabinowitsch and Bagley correction).

In this study, we applied this approach in the full available shear rate range, i.e., at loads M_LL_ = 1.2 kg, M_MIDDLE_ = 5 kg, and M_HL_ = 15 kg. Examples of computations, for the temperature T = 190 °C (A_0_ = 7.824461797, A_1_ = −0.225472825, A_11_ = −0.02811637) are depicted in Figure 11. Viscosity curves determined using the Klein model based on a three-point measurement are shown in relation to plastometric (MFI) measurements and in relation to rheometric (HPCR) measurements (with Rabinowitsch and Bagley correction). These curves correspond well to the results of plastometric tests but also differ from the results of rheometric tests. It should also be noted that the parameters of the Klein model also depend on the choice of measurement points. Furthermore, this equation has limited applications in analytical solutions to polymer processing issues.

### 5.2. On-Line Measurements

The rheological characteristics of the tested material can be determined in the extrusion process based on the measurement of the process output (material flow rate) and pressure measurement in a specialized extrusion die according to the procedure described by Equations (41)–(49).

An experiment was performed using a single screw extruder. A classical screw of diameter D_SCREW_ = 45 mm and the length to diameter ratio (L_SCREW_/D_SCREW_) = 27, and the compression ratio, which is defined as the ratio of the screw channel depth in the feeding section to the screw channel depth in the metering section, equal to CR = H_FEED_/H_METER_ = 2.66, was applied. The extruder was equipped with a specialized extrusion die with a flat (slit) measuring mouthpiece (Figure 3) of dimensions W = 20 mm, H = 2 mm, and L = 80 mm. Various screw rotational speeds, N = 30 rpm, 50 rpm, and 70 rpm, were applied, and the barrel/die temperature profile was set as T_BARREL_ = 180 °C, 180 °C, 190 °C, 190 °C, and T_DIE_ = 190 °C.

The shear rate ${\gamma ˙}_{w}$ at the channel wall, the shear stress at the channel wall $\tau_{w}$, and the viscosity were calculated using Equations (41)–(43). The pressure drop Δp was assumed to be equal to the die pressure p_1_, the volume flow rate was calculated using the melt density, and the shape coefficient f_p_ = f_p_ (H/W) = 0.95 was applied to correct the pressure difference according to Equation (44). With the use of these data, the parameters of the power law were determined using linear regression from Equation (47). And the following model parameters were obtained: the consistency coefficient k = 16,251 Pa·s^n^ and the flow exponent n = 0.244. The results of process measurements and viscosity computations are presented in Table 1.

**Figure 12:**
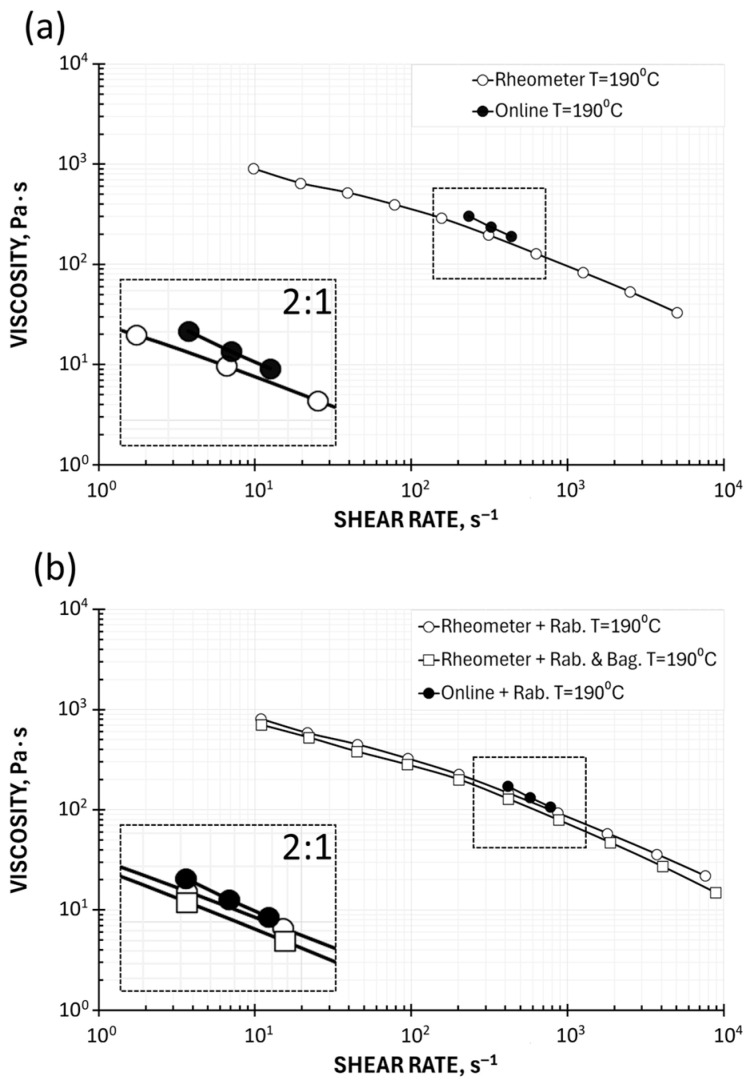
Viscosity curves determined using on-line measurements with local magnifications: (**a**) apparent viscosity in relation to rheometric (HPCR) measurements (apparent viscosity) and (**b**) true viscosity (Rabinowitsch correction) in relation to rheometric (HPCR) measurements (Rabinowitsch and Bagley correction).

The results of on-line viscosity measurements are also depicted in Figure 12 against the background of rheometric viscosity curves. There are visible differences in the results of rheometric and on-line tests; however, despite these discrepancies, it should be noted that the results of on-line measurements well reflect the nature of the dependence of viscosity on shear rate in the achievable shear rate range (from $\dot{\gamma }$ = 200 s^−1^ to $\dot{\gamma }$ = 800 s^−1^).

The accuracy of on-line measurements is subject to errors that increase the viscosity value, which results from the omission of the Bagley correction and the failure to take into account the Rabinowitsch correction. It should be noted, however, that it is possible to apply the Rabinowitsch correction in on-line studies, as was performed in this work (Figure 12b). This reduces the discrepancies between the results of rheometric and on-line measurements (Table 2).

It also seems possible to introduce the Bagley correction into on-line tests, namely, by appropriately designing the measuring die equipped with two flow channels of different lengths, with independent pressure measurements in these channels.

It is important to note that, despite limited accuracy, the production tests have the significant advantage of being conducted under the actual flow conditions of the tested material, rather than under laboratory conditions, as is the case with rheometric and plastometric tests, which do not take into account the processing history of the tested material.

## 6. Conclusions and Future Trends

It is known that the material properties of common polymeric materials (neat polymers) are usually available in appropriate material databases. However, material data, such as rheological data, for materials like polymer blends, polymer composites (including wood plastic composites), and filled plastics, are lacking in these databases.

In the paper, the problem of determining viscosity curves for one of the most widely used advanced polymeric materials, that is, wood plastic composite, was discussed considering the rheological studies in laboratory and production settings, i.e., on-line. Laboratory tests were conducted on the basis of classical rheometric measurements, i.e., High-Pressure Capillary Rheometry (HPCR) and on the basis of Melt Flow Index (MFI) measurements, also including tests based on a limited number of measurement points. Three methods for determining viscosity based on a limited number of measurements were discussed: using the universal Vinogradov–Malkin equation, the two-parameter Ostwald–de Waele power law equation, and the reduced three-parameter Klein logarithmic equation.

This paper shows that viscosity can be determined from Melt Flow Index (MFI) measurements. Furthermore, viscosity curves can be determined based on measurements made at different piston loads. The accuracy of plastometric (MFI) measurements is subject to errors that increase the viscosity value, which results from the use of a short capillary and the omission of the Bagley correction, and the failure to take into account the Rabinowitsch correction. However, it is possible to apply the Rabinowitsch correction in plastometric studies, as was performed in this work, which reduces the discrepancies between the results of rheometric and plastometric measurements. It can be stated that the MFI viscosity is approximately 20–30% higher than the HPCR reference. However, despite significant discrepancies in the results of rheometric and plastometric tests, it should be noted that the results of plastometric measurements reflect the nature of the dependence of viscosity on shear rate, and the achievable shear rate range (from $\dot{\gamma }$ = 10 s^−1^ to $\dot{\gamma }$ = 1000 s^−1^) with a typical set of loads largely covers the shear rate range occurring, e.g., in the polymer extrusion.

The plastometric (MFI) test results based on a limited number of measurements were found to correspond well to the test results based on the measurements over the full available shear rate range, although, as with the full range tests, they differ from the rheometric test results for similar reasons as indicated in the paragraph above. The (MFI) methods based on a limited number of measurement points significantly simplify the study and shorten the study time. The two-point method, based on the power law, is particularly important because this model allows for solving many polymer processing issues analytically, e.g., the pressure flows in extrusion dies and injection molds, as well as the flows in extruders.

It was concluded that the proposed concepts could be useful in engineering practice for quickly assessing the rheological properties of wood plastic composites, as available material databases do not contain such data. Of course, the results obtained using the proposed methods are subject to errors, depending on the type of composite being tested and the range of shear rates and temperatures used. However, they always provide more information about the rheological properties of the processed composite than the Melt Flow Index (MFI), which is not always provided by the manufacturer.

In summary, it is worth noting that calculating the basic rheological problems in polymer processing often does not require the use of advanced computational tools, such as simulation programs. Many problems can be solved based on simple and quick engineering calculations, and the accuracy of such engineering calculations is often only slightly lower than that of calculations performed using advanced simulation programs. Simulation calculations are difficult, expensive, time-consuming, and sometimes lead to erroneous results due to the difficulty of using simulation programs or interpreting the results, as well as the lack of reliable data (material, technological, and geometric) for calculations. Effective use of advanced computational tools requires extensive knowledge of the technology of the problem under consideration, a thorough familiarity with the computational tool, and a solid understanding of rheology and thermodynamics. It is certainly difficult to find specialists with such broad competencies. It is also important to remember that the computational tool should always be selected appropriately for the complexity of the problem under consideration. In the absence of reliable data, especially material data, the use of advanced computational tools is not justified. The long-term goal of this work is to develop an engineering rheological calculation system that will enable rapid calculations of fundamental polymer processing problems with good engineering accuracy. Using such a system should be straightforward and should not require extensive user knowledge. Some elements of this concept can be found in [3,6,72,73].

Rheological studies of wood plastic composites and modeling of wood plastic composite processing, e.g., extrusion or injection molding, require solving some research problems, such as the following:

- A complete rheological analysis of the flow of wood plastic composites requires consideration of possible slippage on the flow channel walls and the yield stress. Slippage and yield stress can lead to plug flow, requiring a fundamental change in the calculation procedures used.
- These issues must also be taken into account when modeling polymer processing, e.g., extrusion or injection molding. Two aspects of slip effects should be considered: rheological and process flow modeling. The effect of slipping can be removed from viscosity measurements by using the Mooney correction, which results in an increase in viscosity since the shear rate decreases as the shear stress remains unchanged. This may have substantial effects on process modeling. On the other hand, considering the slip effects in process modeling results in an increase in the flow rate and pressure decrease. These issues were discussed in [74,75], and, e.g., in [76,77,78,79,80].
- The rheological characteristics of wood plastic composites are generally not available in the material databases. There is no data at all that take into account slip and yield stress, and these data should be obtained in-house, preferably in on-line production conditions.
- The (WPC) material characteristics are highly dependent on the material structure and the size of the fiber or flour. The smaller the size of the filler, the easier and more reliable the measurement. Proper preparation of the material is very important. The results are generally difficult to reproduce. When the material is more homogeneous, the results are more reproducible.
- There is generally no valid thermal data for wood plastic composites, e.g., melting or softening point, heat of fusion, etc. The temperature flow range is narrow, and it is difficult to determine the melting point. Therefore, the concept of no-flow temperature can be used to determine the onset of the flow.
- Due to the lack of rheological data of wood plastic composites in available databases, we propose in-house research methods based on the two-point viscosity curve determination in plastometric (MFI) tests and tests under production conditions, that is, on-line. The two-point method, based on the power law, is quick and easy to implement and allows for solving many polymer processing issues analytically. Of course, the power law model does not describe the low-shear Newtonian plateau, but this is beyond the scope of polymer processing. Production tests, despite limited accuracy, have the significant advantage of being conducted under the actual flow conditions of the tested material, rather than under laboratory conditions, as is the case with rheometric and plastometric tests, which do not take into account the processing history of the tested material.
- A systematic error in plastometric measurements (MFI) was observed, resulting from the use of a short capillary and failure to apply the Bagley correction in the calculations. It seems reasonable to seek a calculation method that takes this systematic error into account.
- A novel concept for on-line testing is proposed, employing a dual-channel measuring die (with different channel lengths) and independent pressure measurement in these channels. This design will eliminate the effect of inlet losses in the measurements and will allow for introducing the Bagley correction into the calculations.

In summary, the concepts and solutions presented in this paper may be applicable to other advanced polymeric materials, i.e., other polymer composites, as well as polymer blends and filled plastics.

## Figures and Tables

**Table 1:** Results of experiment and computations.

Screw Speed	Throughput	Die Pressure	Shear Stress	Shear Rate Apparent	Shear Rate	Viscosity Apparent	Viscosity
N, rpm	G, kg/h	p_D_, MPa	τ_w_, Pa	${\dot{\gamma }}_{a}$, s^−1^	${\dot{\gamma }}_{w}$, s^−1^	η_a_, Pa∙s	η, Pa∙s
30	9.4	5.4	71052.6	232.4	414.1	305.7	171.6
50	13.0	5.8	76315.8	323.2	575.8	236.2	132.5
70	17.6	6.3	82894.7	436.3	777.3	190.0	106.6

**Table 2:** Effect of Rabinowitsch correction on the accuracy of on-line viscosity determination.

Screw Speed	Shear Rate	On-line Viscosity	HPCR Viscosity	Error	HPCR Apparent Viscosity	Error
N, rpm	${\dot{\gamma }}_{w}$	η_on-line_, Pa∙s	η_rheo_, Pa∙s	δ_1_, %	η_a_rheo_, Pa∙s	δ_2_, %
30	414.1	171.6	129.1	24.8	147.3	14.2
50	575.8	132.5	107.7	18.8	123.9	6.6
70	777.3	106.6	91.3	14.4	105.8	0.8

## Data Availability

The original contributions presented in this study are included in the article. Further inquiries can be directed to the corresponding author.
